# A Pharmacist-Managed Hydroxyurea Prescribing Protocol Improves Uptake and Optimization among Patients with Sickle Cell Disease

**DOI:** 10.1155/2024/4753349

**Published:** 2024-05-30

**Authors:** Cameron Roessner, Trudy Sale, Kelsey Uminski, Dawn Goodyear, Natalia Rydz

**Affiliations:** ^1^Southern Alberta Rare Blood and Bleeding Disorders Comprehensive Care Program, Foothills Medical Center, Calgary, AB, Canada; ^2^Pharmacy Services, Foothills Medical Center, Calgary, AB, Canada; ^3^Department of Medicine, Division of Hematology and Hematological Malignancies, University of Calgary, Calgary, AB, Canada

## Abstract

Sickle cell disease (SCD) is a common genetic disorder with potentially serious sequelae that can be effectively treated with hydroxyurea. Despite its favorable benefit-risk profile, hydroxyurea uptake in patients with SCD is low. A pilot study was conducted at the Southern Alberta Rare Blood and Bleeding Disorders (SARBBDs) Comprehensive Care Program between January 2020 and September 2023 to assess the implementation of a pharmacist-led protocol for supporting the uptake of hydroxyurea among eligible patients with SCD and optimizing its dosing. The protocol standardized the prescription, monitoring, dose titration, and patient counselling by a clinic pharmacist. The number of patients enrolled in the SARBBDs program increased from 98 in January 2020 to 168 in 2023. During this period, the proportion of patients on hydroxyurea increased from 37.8% to 62.5%, the proportion of patients on hydroxyurea who were at a maximum tolerated dose (MTD) increased from 35.1% to 63.8%, and the average hemoglobin F level increased from 13.9% to 19.7%. The mean time to reach MTD was 10 months and required eight pharmacist interventions, six laboratory assessments, and three dose increases. Hydroxyurea continuation rates were high, with most discontinuations resulting from loss to follow-up or transition to a transfusion management strategy. This real-world pilot study demonstrated that implementation of a pharmacist-led prescribing and monitoring protocol nearly doubled hydroxyurea uptake and achievement of MTD in patients with SCD managed in a rare blood disorders clinic.

## 1. Introduction

Sickle cell disease (SCD) is the most prevalent genetic disorder worldwide. While the majority of people with SCD are from sub-Saharan Africa, it is also a health concern in North American populations with an estimated incidence of 1 in 365 African-Americans and 1 in 16,300 Hispanic Americans [1, 2]. SCD is associated with potentially serious sequelae including pain crises, acute chest syndrome, infections, organ damage, and musculoskeletal complications [1, 3–5]. In addition to the physical morbidity of SCD, the disease can have a substantial negative impact on patients' quality of life, [3, 6] psychosocial health, [3, 6] and life expectancy, [7] and is associated with high healthcare resource utilization [3].

Hydroxyurea is an inexpensive oral medication that has demonstrated benefits in the management of SCD [3]. It has been shown to reduce painful episodes of SCD by 44% [8] and mortality by 40% [9]. Other benefits include reductions in hospital admissions, episodes of acute chest syndrome, and the need for blood transfusions [8]. It is generally safe and well tolerated, with limited short-term toxicities such as nail color changes, dose-dependent cytopenias, and gastrointestinal symptoms, with no known long-term complications [9–11]. Importantly, there is no evidence to suggest the development of resistance over time, making long-term treatment possible [10]. Hydroxyurea is recommended by evidence-based guidelines [12, 13] and it is currently the only approved disease-modifying treatment for SCD in Canada. It is conveniently dosed once daily by oral administration, making it safer and more efficient and cost-effective than chronic blood transfusion [1].

Despite its favorable benefit-risk profile and cost-effectiveness, the uptake of hydroxyurea is poor. According to database reviews in the United States (U.S.), only 11–23% of eligible individuals are on hydroxyurea [14, 15] with the highest rates of uptake in pediatric patients compared to older adults [15]. There are myriad patient-related factors that could account for the low uptake of hydroxyurea including the poor perception of SCD as a life-shortening disease, misconceptions about hydroxyurea efficacy and safety, not being offered treatment, and not having concerns addressed by healthcare providers (HCPs) [11, 16, 17]. In addition, there are drug- and health system-related factors such as the frequent lab evaluations to achieve a maximally tolerated dose (MTD) and for safety monitoring, and provider hesitancy to prescribe hydroxyurea [16].

Multidisciplinary rare blood disorder clinics are well-positioned to optimize the uptake of hydroxyurea among patients with SCD. In Alberta, Canada, pharmacists can independently prescribe a wide variety of medications with approval from the provincial pharmacy regulatory body [17]. The objective of this study was to assess the implementation of a pharmacist-led protocol for supporting patients with SCD, thereby increasing the uptake of hydroxyurea treatment for eligible patients, and optimizing hydroxyurea dosing in Alberta.

## 2. Methods

### 2.1. Study Setting

The study was conducted at the Southern Alberta Rare Blood and Bleeding Disorders (SARBBDs) Comprehensive Care Program.

### 2.2. Study Population

Patients aged ≥18 years with a diagnosis of SCD (hemoglobin S homozygous (HbSS), heterozygous (Hb SC), or sickle beta 0 thalassemia (S*β*° Thal)), and who were registered with the SARBBDs from January 1, 2020, to September 20, 2023, were included. There were no exclusion criteria for the study.

### 2.3. Study Design

To assess the barriers limiting the use of hydroxyurea, a simple survey (see Supplementary Material) was e-mailed to patients registered with the SARBBDs and who had provided permission to be contacted by e-mail. The survey was conducted using REDCap, a secure web application for online surveys and databases. Patients were asked to respond to multiple choice and short answer questions addressing their understanding of and history with hydroxyurea, reasons (if any) for discontinuation and/or declining to start treatment, side effects (if any) with hydroxyurea in the past, concerns with side effects, and access through drug coverage.

To standardize the prescription, monitoring, dose titration, and support for patients starting hydroxyurea, a clinic protocol for pharmacist-led management was developed. The role of the pharmacist (C.R.) included hydroxyurea bloodwork monitoring and dose titration to the MTD; communication with patients regarding side effects, adherence, and bloodwork reminders; navigating drug coverage (with involvement from social work to assess drug coverage issues and navigate enrollment for existing coverage, as needed); communication with community pharmacies; assisting with compounding access; checking for drug interactions and contraindications; and counselling regarding the use of reliable contraception while taking hydroxyurea. These activities were completed within a part-time role of the pharmacist.

The impact of implementing the pharmacist-led prescribing protocol on the proportion of patients on hydroxyurea and the proportion of patients achieving MTD and the average hemoglobin F (HbF) levels were evaluated at the end of each year during the study period.

### 2.4. Hydroxyurea Prescribing and Monitoring Protocol

The hydroxyurea prescribing and monitoring protocol used in this study was adapted from the CanHaem consensus statement [12] and the National Heart, Lung, and Blood Institute's (NHLBI) Sickle Cell Guidelines [18] and individualized for each patient, as appropriate. Hydroxyurea was strongly recommended for all patients with HbSS and Hb S*β*° Thal. For those with Hb SC, we encouraged hydroxyurea therapy for those with chronic complications or acute severe complications (such as VOC or ACS) who also had a hematocrit of <0.32–0.35 L/L. This practice is aligned with other centers and local guidelines [12, 19].

#### 2.4.1. Patient Education and Counselling

CanHaem and American Society of Hematology hydroxyurea patient handouts and/or a SARBBDs presentation were provided to and reviewed with the patients by a nurse coordinator (T.S.). The need for routine blood work for monitoring, potential teratogenic effects of the medication, the need for reliable contraception while on treatment, and contraindications in pregnancy and breastfeeding were reviewed with patients.

#### 2.4.2. Initial Prescription

Patients were initiated at a dose of 500 mg hydroxyurea orally (PO) for one week to limit gastrointestinal symptoms, and then increased to a starting dose of 1,000 mg PO daily. For patients with a body mass of <60 kg or with a glomerular filtration rate (GFR) < 60 mL/min, lower doses were considered.

#### 2.4.3. Monitoring

Baseline monitoring included complete blood count (CBC), reticulocyte count, HbF level, renal and liver function, and a pregnancy test (when appropriate). While titrating the hydroxyurea dose, these parameters were monitored every 2–4 weeks. When the hydroxyurea dose was stable, the abovementioned assessments were conducted every 3 months. The CBC reassessment frequency could be increased to weekly if hydroxyurea was withheld due to cytopenia. Adherence was reviewed at each clinic visit.

#### 2.4.4. Titration of Hydroxyurea Dose

If desired changes in blood work (e.g., increased mean corpuscular volume (MCV), decreased neutrophil count, or increased HbF) were minimal or absent, the pharmacist first reviewed the hydroxyurea dose with the patient, assessed adherence, and assisted with finding strategies for adherence, and then adjusted the hydroxyurea dosing to achieve the patient's MTD (defined as the maximum dose that maintained neutrophils at ≤1.5 × 10^9^/L, platelets at >80 × 10^9^/L, and overall hemoglobin at >50 g/L). MTD was selected for this study instead of fixed dosing since there is evidence to support higher efficacy when hydroxyurea is titrated to MTD than fixed dosing, which usually involves a lower albeit “clinically therapeutic” dose [20]. When hydroxyurea dose increases were necessary, the dose was increased every 2–4 weeks by 500 mg every one or two days, until a maximum dose of 35 mg/kg was reached. If neutrophils, platelets, overall hemoglobin, or reticulocytes dropped to <1.0 × 10^9^/L, <80 × 10^9^/L, <50 g/L, or <80 × 10^9^/L, respectively, hydroxyurea was held until recovery, monitored weekly, and resumed at a reduced dose by ∼5 mg/kg per day. A six-month period at the MTD was required prior to considering discontinuation due to treatment failure or considering switching to a transfusion/phlebotomy program. A lack of increase in MCV and/or HbF was not considered an indication to discontinue therapy. As per protocol, hydroxyurea should have been continued during hospitalizations or illness except in cases of reticulocytopenia or overwhelming infection.

### 2.5. Analysis

Descriptive statistics of the study participants were tabulated and presented using means and ranges for continuous variables and percentages for discrete variables.

### 2.6. Ethical Considerations

A pRoject Ethics Community Consensus Initiative (ARECCI) Ethics Screening Tool, developed by Alberta Innovates-Health Solutions (AIHS), was used. Using this tool, the study was determined to be a quality improvement project with minimal risk. The ARECCI Ethics Guidelines for Quality Improvement and Evaluation Projects were followed to ensure the study adhered to all ethical standards.

## 3. Results

There were 98 eligible patients registered with the SARBBDs as of January 1, 2020, who were included in the pharmacist-led prescribing study; this number increased to 168 by September 20, 2023 (Table 1). More than half of the patients were female (56.9–60.2%), and most patients were HbSS (67.3–73.2%) with few Hb S*β*° Thal patients (2.4–3.7%) (Table 1).

**Figure 1:**
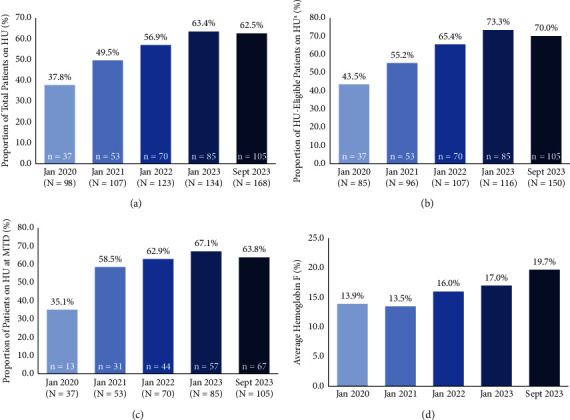
Impact of the pharmacist-led prescribing and monitoring protocol on (a) proportion of total patients on hydroxyurea, (b) proportion of total hydroxyurea-eligible patients on hydroxyurea, (c) proportion of patients on hydroxyurea who were on the maximum tolerated dose, and (d) average hemoglobin F % in each year of the evaluation period. Patients not on an exchange/transfusion program. HU = hydroxyurea and MTD = maximum tolerated dose.

The proportion of patients on hydroxyurea increased from 37.8% (*n* = 37/98) to 62.5% (*n* = 105/168) with pharmacist-led prescribing during this time period (Figure 1(a)). The proportion of patients on hydroxyurea increased over time for patients with HbSS and Hb SC genotypes and remained stable for the small subgroup of Hb S*β*° Thal patients (Table 2). When only patients eligible for hydroxyurea (i.e., patients not on an exchange/transfusion program) were considered in September 2023, the proportion of patients on hydroxyurea increased to 70.0% (*n* = 105/150) (Figure 1(b)). In September 2023, the median daily dose of hydroxyurea was 1,500 mg in the overall cohort and in patients with HbSS. In patients with Hb SC and Hb S*β*° Thal, the median daily dose was 1,500 mg from Monday to Friday and 1,000 mg on Saturday and Sunday.

Among patients on hydroxyurea, 35.1% (*n* = 13/37) and 63.8% (*n* = 67/105) were at the MTD in January 2020 and September 2023, respectively (Figure 1(c)). The average HbF level increased from 13.9% in January 2020 to 19.7% in September 2023 (Figure 1(d)). When separated into subgroups based on sickle hemoglobin genotype, the average HbF level increased from 15.8% to 22.7% in patients with HbSS, 2.6% to 9.1% in patients with Hb SC, and remained relatively stable (21.0% to 18.9%, with a peak of 25.1%) in the small subgroup of patients with Hb S*β*° Thal (see Supplementary Material, Figure S1). During the evaluation period, there were monthly averages of 1.9 new patients starting on hydroxyurea, 5.3 hydroxyurea dose adjustments (mostly increases, with some interruptions or reductions), 29.4 pharmacist interactions (education and follow-up), and 16.7 patient laboratory visits. It took a mean of 10 months from the initiation of hydroxyurea to reach MTD, requiring an average of eight pharmacist interactions, six laboratory appointments, and three dose increases.

Out of 133 total patients exposed to hydroxyurea, 28 stopped treatment during the evaluation period. Most patients who stopped hydroxyurea were lost to follow-up (*n* = 10) or stopped to undergo transfusions (*n* = 7). Two patients stopped treatment to undergo curative bone marrow transplantation, and others stopped treatment because of side effects including gastrointestinal symptoms (bloating/weight gain, *n* = 2; diarrhea, *n* = 2), oral toxicity (mucocutaneous pain, *n* = 2), cutaneous ulceration (malleolus ulcer, *n* = 2), and gangrene (dry gangrene of toes not previously reported in SCD, *n* = 1).

## 4. Conclusions

This pilot study demonstrated that implementation of a pharmacist-managed prescribing protocol improved hydroxyurea uptake and achievement of MTD in patients managed in a rare blood disorders clinic. The proportion of eligible patients on hydroxyurea nearly doubled over a period of approximately 3.5 years, and a similar increase in the proportion of patients achieving MTD was achieved.

Together, these observations suggest that barriers to hydroxyurea utilization, including misinformation, concerns about side effects, frequency of lab monitoring, and medication access, can be overcome by a variety of interventions tailored to individual clinic settings and resources. In our study, a pharmacist dedicated to working in a rare blood disorders clinic implemented a patient education and monitoring protocol, but the model could be extended to other HCPs including nurses/nurse practitioners specialized in rare blood disorders. These allied health professionals may be more accessible to patients than hematologists, and our experience anecdotally suggests that patients were enthusiastic about pharmacist involvement in their ongoing care. Furthermore, the framework of this pilot program may have broader applicability on a global scale, including in resource-limited regions where access to a hematologist may be a particularly important barrier to care.

The promising observations from the present study are aligned with reports from other settings that have implemented a variety of interventions aimed at increasing the uptake of hydroxyurea. One community hospital in Brampton, Ontario, Canada implemented a new adult and pediatric SCD clinic with targeted education for patients and HCPs [21]. Hydroxyurea uptake increased significantly from 41% to 60% from 2017 to 2019 (*p* < 0.001), and the annual admission rate for SCD-related acute pain syndromes fell from 90% to 75% (*p*=0.010). Moreover, the average length of hospital stay fell significantly from 3.5 to 2.9 days (*p*=0.039).

In a multidisciplinary blood disorders clinic in Ohio, U.S., implementing a change in consenting practices increased hydroxyurea uptake among individuals with SCD aged 2–20 years by 158% (*p* < 0.001) over a 1-year period from 2012 to 2013 [11]. The consenting process was expanded from a generic consent form to the provision of both written and verbal information on how hydroxyurea works, its safety and tolerability profile, and its benefits, using plain language and visual descriptions. The information specifically addressed patient barriers such as inaccurate perceptions of side effects and personalized discussions about other concerns.

A tertiary hematology clinic in Florida developed a 15-minute educational video for patients with SCD, outlining the benefits and risks of hydroxyurea as well as patient experiences while taking the medication [22]. The video encouraged patients to ask their HCP about whether hydroxyurea was appropriate for their condition, and was accompanied by a pamphlet written in plain language. The materials were piloted in 58 patients with SCD and showed that 40% discussed hydroxyurea with their HCP after viewing the video and an additional 45% had intentions of doing so in the next month. Thus, passive delivery of education may also have a beneficial impact on hydroxyurea uptake.

In addition, the findings of an observational study by Pecker et al. suggest that targeting educational interventions to patients with SCD and their families in the week after an urgent care visit or hospitalization could be an optimal time to encourage hydroxyurea initiation [17]. The intervention consisted of a hematologist-led discussion on the benefits and risks of hydroxyurea, a 15-minute video showcasing patient experiences with hydroxyurea treatment, and a direct offer to start hydroxyurea. Following the intervention, 55% of patients had initiated hydroxyurea, which was significantly higher than a control group of patients who did not receive the intervention (20%; *p*=0.0004). Two years after implementing the intervention, the rate of hydroxyurea uptake among patients presenting to the emergency department increased from 56% to 80% (*p*=0.0069).

Despite the encouraging results of this pilot study and others, uptake of hydroxyurea among eligible patients with SCD remains suboptimal. Indeed, in our study, 30% of patients with SCD not receiving transfusions are still not taking hydroxyurea. Similarly, after Smith et al. implemented a more targeted consenting procedure, 55% of eligible patients were not on the drug, [11] suggesting additional measures are needed to further improve acceptability.

This study has limitations. This was a noninterventional observational study and as such, the increase in hydroxyurea uptake cannot be attributed solely to the pharmacist-led intervention since other factors could have also influenced uptake. As is the case with any single-center study, it is unclear whether the demographics and characteristics of the patient population included are generalizable to the broader Canadian or North American population. In addition, clinical data on hard outcomes such as acute pain episodes or hospitalizations were not collected, and patient-reported outcomes were not completed to assess the acceptability of the program. Furthermore, this study was conducted in Alberta, Canada, where pharmacists are able to prescribe a wide variety of medications. It should be noted that pharmacists do not have the same prescribing capabilities in all jurisdictions. Nonetheless, the study design reflects a real-world clinical setting and suggests that the implementation of a pharmacist-led hydroxyurea prescribing protocol is feasible in the setting of a multidisciplinary rare blood disorders clinic.

In conclusion, this real-world pilot implementation of a pharmacist-managed hydroxyurea prescribing protocol for patients with SCD was feasible and resulted in a nearly doubling of hydroxyurea uptake and a similar increase in the proportion of patients achieving MTD. Together with the observed improvement in biochemical outcomes, improvements in long-term clinical outcomes and healthcare utilization would be expected based on the increased uptake of a safe disease-modifying treatment with demonstrated effectiveness [18].

## Figures and Tables

**Table 1:** Patient characteristics.

	January 2020 (*N* = 98)	January 2021 (*N* = 107)	January 2022 (*N* = 123)	January 2023 (*N* = 134)	September 2023 (*N* = 168)
Age, mean (SD), years	35.8 (11.7)	35.9 (11.4)	35.8 (11.3)	36.3 (10.9)	35.5 (11.1)
*Gender*					
Male, *n* (%)	38 (38.8)	42 (39.3)	52 (42.3)	56 (41.8)	67 (39.9)
Female, *n* (%)	59 (60.2)	64 (59.8)	70 (56.9)	77 (57.5)	100 (59.5)
Nonbinary, *n* (%)	1 (1.0)	1 (0.9)	1 (0.8)	1 (0.7)	1 (0.6)
*Sickle hemoglobin genotype*					
HbSS, *n* (%)	71 (72.4)	72 (67.3)	86 (69.9)	98 (73.1)	123 (73.2)
Hb SC, *n* (%)	24 (24.5)	31 (29.0)	33 (26.8)	32 (23.9)	41 (24.4)
Hb S*β*° Thal, *n* (%)	3 (3.1)	4 (3.7)	4 (3.3)	4 (3.0)	4 (2.4)

**Table 2:** Proportion of patients on hydroxyurea per year by sickle hemoglobin genotype.

	January 2020 (*N* = 98)	January 2021 (*N* = 107)	January 2022 (*N* = 123)	January 2023 (*N* = 134)	September 2023 (*N* = 168)
HbSS, *N*	71	72	86	98	123
Hydroxyurea use, *n* (%)	30 (42.3)	39 (54.2)	52 (60.5)	65 (66.3)	80 (65.0)
Hb SC, *N*	24	31	33	32	41
Hydroxyurea use, *n* (%)	5 (20.8)	11 (35.5)	15 (45.5)	17 (53.1)	22 (53.7)
Hb S*β*° Thal, *N*	3	4	4	4	4
Hydroxyurea use, *n* (%)	2 (66.7)	3 (75.0)	3 (75.0)	3 (75.0)	3 (75.0)

## Data Availability

The data used to support the findings of this study are included within the article.

## Supplementary Materials

Supplementary Materials The SARBBDs Hydroxyurea Patient Survey aims to understand the various barriers that limit hydroxyurea (HU) use among patients with sickle cell disease (SCD). The survey investigates patient experiences with HU, including their understanding of HU benefits, past side effects and reasons for stopping HU (if applicable), concerns about side effects (including cancer risk, family planning, and bloodwork requirements), medication access issues, and patient confidence in their knowledge of HU and SCD for informed treatment decisions. The SARBBDs Hydroxyurea Prescribing and Monitoring Protocol provides a framework for prescribing and managing hydroxyurea treatment in sickle cell disease patients. Adapted from established guidelines, the protocol emphasizes patient education about hydroxyurea's benefits, side effects, the need for contraception, and the importance of routine bloodwork monitoring. It outlines initial dosage, considerations for dose adjustments, and monitoring parameters based on blood test results. The protocol stresses the importance of patient adherence and aims to determine the maximum tolerated dose (MTD) for each individual patient to optimize treatment outcomes.
