# Supplementary material for: A Pharmacist-Managed Hydroxyurea Prescribing Protocol Improves Uptake and Optimization among Patients with Sickle Cell Disease
Source: Adv Hematol. 2024 May 30;2024:4753349. doi: 10.1155/2024/4753349 (PMC11192600; doi:10.1155/2024/4753349)
Supplement: Supplementary Materials — The SARBBDs Hydroxyurea Patient Survey aims to understand the various barriers that limit hydroxyurea (HU) use among patients with sickle cell disease (SCD). The survey investigates patient experiences with HU, including their understanding of HU benefits, past side effects and reasons for stopping HU (if applicable), concerns about side effects (including cancer risk, family planning, and bloodwork requirements), medication access issues, and patient confidence in their knowledge of HU and SCD for informed treatment decisions. The SARBBDs Hydroxyurea Prescribing and Monitoring Protocol provides a framework for prescribing and managing hydroxyurea treatment in sickle cell disease patients. Adapted from established guidelines, the protocol emphasizes patient education about hydroxyurea's benefits, side effects, the need for contraception, and the importance of routine bloodwork monitoring. It outlines initial dosage, considerations for dose adjustments, and monitoring parameters based on blood test results. The protocol stresses the importance of patient adherence and aims to determine the maximum tolerated dose (MTD) for each individual patient to optimize treatment outcomes. [file 4753349.f1.docx]

# Supplementary Material

**The SARBBD Hydroxyurea Patient Survey**

Are you taking hydroxyurea (HU)?

Yes – Answer PART A

No – Answer PART B

Part C – ALL

PART A

1. Why was HU prescribed for you? (select all that apply)
   1. Frequent pain crises
   2. Chest syndrome
   3. History of stroke
   4. Organ/bone damage from sickle cell disease
   5. I don’t know
   6. Other

If other, explain: ____________

1. Do you think HU is helping you?

Yes/No

2a. If so, how? ____________

1. On a scale of 1-10 (10 being very satisfied), how do you feel about the HU teaching and counselling you received prior to starting it?

3a. Any comments? ____________

1. Would you recommend HU to another patient with sickle cell disease?

Yes/No

4a. Why or why not? ____________

1. In an average week:

5a. How many doses of HU do you miss?

5b. What are the reasons? (select all that apply)

- 1. I forget to take it
  2. Don’t feel like taking it
  3. Don’t want/have experienced side-effects
  4. Lost the prescription
  5. Ran out and didn’t pick up a new supply
  6. Ran out and didn’t get a new prescription
  7. Could not afford it
  8. Trouble swallowing the capsules
  9. Feel like I don’t know enough about the medication
  10. Other

If other, describe: ____________

PART B

1. Why are you not on HU?
   1. It has never been mentioned/offered to me
   2. I have been given a prescription but I didn’t/forgot fill it
   3. I took it but then stopped.
   4. Other:____________
2. If you have never been on HU, what are the barriers for not taking it? Rank all on a scale of 1-5 with 1 being not a factor and 5 being a major barrier.
   1. Concerns about cost
   2. Concerns about side-effects
   3. Didn’t think I needed it
   4. Concerns about cancer risk
   5. Wanting to start a family
   6. Concerns about needing regular bloodwork
   7. Don’t see the point of taking it
   8. Heard from a friend/family member that it was a bad drug/has side-effects
   9. Other____________
   10. N/A
3. If you have been on HU in the past, but are not anymore, why?
   1. Kept forgetting to fill a prescription
   2. Didn’t see an improvement/or see the point,
   3. Side-effects: what were they? ____________
   4. Cost
   5. Trouble swallowing the pill
   6. Didn’t like to do the blood work.

PART C

1. Do you feel that you know enough about HU to make a good decision about taking it or not taking it?

Yes/No

1. Do you feel that you know enough about sickle cell disease to make a good decision about how to manage it?

Yes/No

1. Are you comfortable asking questions about your treatment plan with HU?

Yes/No

Do you have any other comments? ____________

**The SARBBD Hydroxyurea Prescribing and Monitoring Protocol**

Adapted from the CanHaem Consensus statement (2015)^12^ and the NHLBI Sickle Cell Guidelines (2014)^18^. To be individualized for each patient, as appropriate.

Patient Education and Counselling:

- Provide and review with patient any of the following: the CanHaem and ASH hydroxyurea patient handouts, SARBBD presentation
- Review the need for routine blood work monitoring
- Review the potential teratogenic effects, and need for reliable contraception
- Review the contraindication in pregnancy and breastfeeding

Initial Prescription:

- Ensure drug coverage
- Start at a dose of 500 mg PO daily for one week, then increase to starting dose of 1,000 mg PO daily
  - Consider lower doses those with weight of <60 kg, or with GFR <60 mL/min

Monitoring:

- Baseline: CBC, reticulocyte count, HbF level, renal and liver function, ± pregnancy test
- While titrating hydroxyurea dose: CBC, reticulocyte count, HbF level, renal and liver function every 2-4 weeks. If the hydroxyurea is held for cytopenias, consider reassessing CBC weekly
- On a stable dose of hydroxyurea: CBC, reticulocyte count, HbF level, renal and liver function every 3 months
- At each clinic visit, review adherence

Titration of hydroxyurea dose, based on the monitoring bloodwork:

- If no or minimal changes on bloodwork (increased MCV, decreased neutrophil count or increased HgF), FIRST:
  - Review the hydroxyurea dose with patient
  - Assess adherence and assist with finding strategies for compliance
    THEN:
- Adjust hydroxyurea dosing to achieve the patients maximum tolerated dose, defined as the maximum dose that maintains neutrophils ≥1.5 x 10^9^/L, platelets >80 x 10^9^/L, and overall hemoglobin >50 g/L
- When increasing hydroxyurea, increase by 500 mg daily or every second day until a maximum dose of 35 mg/kg
- If neutrophils <1.0 x 10^9^/L, platelets <80 x 10^9^/L, overall hemoglobin <50 g/L, or reticulocytes <80 x 10^9^/L, hold hydroxyurea until recovery, monitor weekly and then resume hydroxyurea at a reduced dose by ~5 mg/kg per day
- A 6-month period at the MTD is required prior to considering discontinuation due to treatment failure and consider switching to a transfusion/phlebotomy program as indicated
- A lack of increase in MCV and/or HbF is not an indication to discontinue therapy
- Hydroxyurea therapy should be continued during hospitalizations or illness unless there is reticulocytopenia or overwhelming infection
